# Vessel-Specific Differences in Fractional Flow Reserve Among Intermediate Coronary Lesions

**DOI:** 10.3390/jcm15093465

**Published:** 2026-05-01

**Authors:** Victor Weerts, Cedric Davidsen, Mathieu Lempereur, Patrick Marechal, Laurent Davin, Christophe Martinez, Patrizio Lancellotti

**Affiliations:** Groupe Interdisciplinaire de Génoprotéomique Appliquée (GIGA) Cardiovascular Sciences, Centre Hospitalier Universitaire (CHU) Sart Tilman, Department of Cardiology, University of Liège Hospital, 4000 Liège, Belgium; vweerts@chuliege.be (V.W.); cedric.davidsen@chuliege.be (C.D.); mathieu.lempereur@chuliege.be (M.L.); pmarechal@chuliege.be (P.M.); ldavin@chuliege.be (L.D.);

**Keywords:** coronary physiology, fractional flow reserve, chronic coronary artery disease, ischemic heart disease

## Abstract

**Background/Objectives**: Fractional flow reserve (FFR) is the reference standard for assessing the functional significance of intermediate coronary stenoses and guiding revascularization. Although a universal ischemic threshold is applied to all epicardial vessels, potential physiological differences between coronary territories remain insufficiently explored. The aim of this study was to evaluate whether the functional significance of intermediate coronary stenoses differs according to coronary artery and to assess the clinical outcomes of FFR-guided deferral across coronary territories. **Methods**: This single-center retrospective study included patients who underwent single-vessel FFR assessment for angiographically intermediate lesions between 2019 and 2022. Patients with left main disease or multivessel physiological assessment were excluded. Clinical characteristics, FFR values, and long-term outcomes were analyzed according to the investigated coronary artery. Major adverse cardiovascular events (MACE) were defined as a composite of death, myocardial infarction, and urgent revascularization. **Results**: A total of 310 patients (corresponding to 310 coronary arteries) were included: 211 LAD, 68 RCA, and 31 LCX lesions. Overall, 18.7% of lesions had a positive FFR (≤0.80). The only variable identified in univariable analysis as being associated with FFR positivity was the coronary artery evaluated (*p* < 0.001). Positive FFR values were observed in 24.6% of LAD lesions, compared with 8.8% in the RCA and none in the LCX. Among patients with negative FFR for whom revascularization was deferred, five-year MACE-free survival was similar across coronary territories (*p* = 0.12). **Conclusions**: The functional significance of intermediate coronary stenoses varies according to the coronary territory, with LAD lesions more frequently reaching ischemic thresholds. However, deferral of revascularization based on negative FFR is associated with favorable long-term outcomes across all vessels, supporting a vessel-specific physiological interpretation of coronary stenoses.

## 1. Introduction

Coronary artery disease is a leading cause of morbidity and mortality worldwide, with ischemic heart disease representing the most common clinical manifestation [1,2]. Although coronary angiography remains the cornerstone for anatomical assessment, it provides limited information regarding the functional significance of intermediate coronary stenoses. As a result, decisions based solely on angiographic severity may lead to both under- and overtreatment. Over the past two decades, the integration of coronary physiology into clinical decision-making has transformed the management of stable coronary artery disease.

Fractional flow reserve (FFR), defined as the ratio of distal coronary pressure to aortic pressure during maximal hyperemia, has become the reference standard for assessing the hemodynamic relevance of coronary lesions [3,4]. Randomized trials have consistently demonstrated that FFR-guided revascularization improves clinical outcomes while reducing unnecessary procedures, using a universal ischemic threshold of 0.80 [5,6]. However, the widespread adoption of FFR has been accompanied by the implicit assumption that the physiological behavior of coronary stenoses is similar across all epicardial vessels. In daily practice, lesions with comparable angiographic severity may yield markedly different FFR values depending on their anatomical location. This observation suggests that vessel-specific factors, such as myocardial territory supplied, coronary dominance, and microvascular characteristics, may influence the physiological impact of a given stenosis. Emerging imaging and physiological studies have demonstrated that the amount of subtended myocardial mass is a major determinant of coronary flow and pressure gradients [7,8]. Since the left anterior descending artery (LAD) typically supplies the largest proportion of left ventricular myocardium, lesions in this vessel may be more likely to produce ischemia than anatomically similar lesions in the right coronary (RCA) or circumflex (LCX) arteries. Despite this plausible mechanistic framework, comparative data on FFR behavior across different coronary territories remain limited in real-world clinical settings.

The aim of the present study was therefore to compare FFR values among the major coronary arteries in patients with angiographically intermediate lesions and to evaluate the prognostic implications of vessel-specific physiological differences.

## 2. Materials and Methods

### 2.1. Study Design and Population

This single-center retrospective observational study was conducted at Liège University Hospital and included all consecutive patients who underwent invasive coronary physiology assessment using fractional flow reserve (FFR) between 1 January 2019 and 31 December 2022.

**Figure 1:**
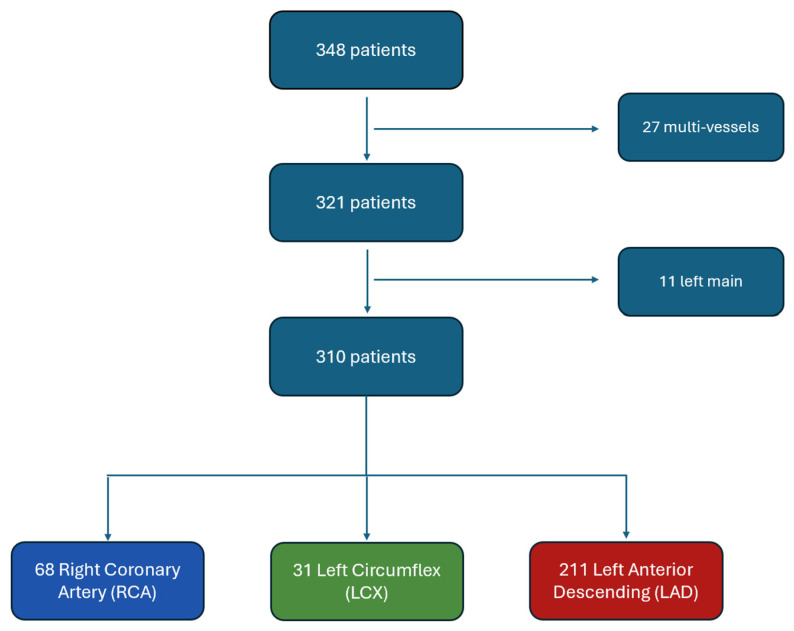
Study flowchart. A total of 348 patients were screened. Twenty-seven patients with multivessel FFR assessment were excluded. Among the remaining patients, those with isolated left main assessment were also excluded. The final study population consisted of 310 patients with single-vessel FFR assessment, corresponding to 310 coronary arteries (211 LAD, 68 RCA, and 31 LCX).

FFR measurements were performed in angiographically intermediate lesions when angiography alone was insufficient to guide revascularization. Intermediate lesions were defined by visual estimation of diameter stenosis by the operator during coronary angiography. These typically corresponded to a stenosis severity of approximately 40–70%. Patients presenting with chronic coronary syndromes as well as those admitted for acute coronary syndromes, in whom a non-culprit lesion was assessed, were eligible for inclusion. Patients were excluded if they had left main coronary artery disease or if multivessel coronary physiology assessment was performed during the same procedure, as attribution of clinical outcomes to a single coronary artery would not have been possible. Patients with incomplete clinical or follow-up data were also excluded. Among the 348 patients initially screened, 27 patients who underwent multivessel physiological assessments and 11 patients with left main disease were excluded. The final study population therefore consisted of 310 patients, each with a single coronary artery assessed by FFR (211 left anterior descending arteries [LAD], 68 right coronary arteries [RCA], and 31 left circumflex arteries [LCX]) (Figure 1).

### 2.2. FFR Measurement

FFR measurements were performed using a pressure-sensor guidewire in accordance with contemporary clinical practice. After pressure equalization at the coronary ostium, the guidewire was advanced distal to the target lesion. Maximal hyperemia was induced using either intravenous or intracoronary adenosine at the discretion of the operator. FFR was calculated as the ratio of mean distal coronary pressure to mean aortic pressure during stable hyperemic conditions. All measurements were obtained with the patient in the supine position, and careful verification of pressure drift was systematically performed at the end of each recording. An FFR value of 0.80 or less was considered indicative of functionally significant myocardial ischemia and used as the threshold for deferral or performance of coronary revascularization.

### 2.3. Data Collection and Follow-Up

Baseline demographic characteristics, cardiovascular risk factors, comorbidities, laboratory parameters, angiographic findings, and pharmacological treatments were collected retrospectively from electronic medical records. Follow-up data were obtained from hospital databases and outpatient clinic visits. Completeness of data was verified for all variables included in the analysis, and no missing values were identified; therefore, no imputation method was required. The primary clinical endpoint was the occurrence of major adverse cardiovascular events (MACE), defined as a composite of all-cause mortality, myocardial infarction, and urgent coronary revascularization. Follow-up was completed on 15 November 2023.

### 2.4. Statistical Analysis

Continuous variables are expressed as mean ± standard deviation, whereas categorical variables are presented as numbers and percentages. Comparisons of FFR values between coronary arteries were performed using the Kruskal–Wallis test. Univariable logistic regression analyses were used to identify clinical and procedural predictors of a positive FFR value, expressed as odds ratios with corresponding 95% confidence intervals. The primary variable of interest—coronary vessel territory—was pre-specified based on the study hypothesis that FFR positivity differs between epicardial coronary arteries. Additional clinical and demographic variables were included in univariable analyses as covariates selected based on a priori clinical relevance rather than as exploratory variables. When standard logistic regression models could not be computed due to the absence of events in one subgroup, Firth’s penalized logistic regression was applied to address issues of complete or quasi-complete separation and reduce small-sample bias. A multivariable Firth’s penalized logistic regression model was then constructed to assess independent predictors of FFR positivity after adjustment for potential confounders, including vessel territory, age, sex, diabetes, hypertension, and smoking history. These covariates were selected a priori based on established cardiovascular risk factors and clinical relevance. Prior to model construction, collinearity among candidate covariates was evaluated using variance inflation factor (VIF) analysis; no variable exceeded a VIF of 5, indicating the absence of problematic multicollinearity. Time-to-event analyses were performed using Kaplan–Meier survival curves, and differences between groups were assessed using the log-rank test. Univariable Cox proportional hazards regression was additionally performed to estimate hazard ratios for MACE across coronary territories. A two-sided *p* value of less than 0.05 was considered statistically significant. A post hoc power analysis was performed using the pwr package in R, showing an observed power of 0.545 for the survival comparison. All statistical analyses were conducted using R version 4.5.1 (R Core Team, 2025) within RStudio version 2025.09.1 (Posit Software, PBC, Boston, MA, USA), and graphical representations were generated using the ggplot2 package version 4.0.2.

## 3. Results

### 3.1. Baseline Characteristics

A total of 310 patients were included in the final analysis. Among them, 58 patients (18.7%) had a positive FFR value (≤0.80), while 252 patients (81.3%) had a negative FFR value (>0.80). FFR assessment was performed most frequently in the left anterior descending artery (211 cases, 68.1%), followed by the right coronary artery (68 cases, 21.9%) and the left circumflex artery (31 cases, 10.0%). Baseline demographic and clinical characteristics according to FFR status are summarized in Table 1. 

No significant differences were observed between patients with positive and negative FFR values with respect to age, sex, body mass index, cardiovascular risk factors, renal function, previous myocardial infarction, previous revascularization, or left ventricular ejection fraction. Similarly, background pharmacological treatments, including antiplatelet agents, beta-blockers, renin–angiotensin system inhibitors, and statins, were not associated with the likelihood of a positive FFR result.

### 3.2. FFR Distribution According to Coronary Artery

**Figure 2:**
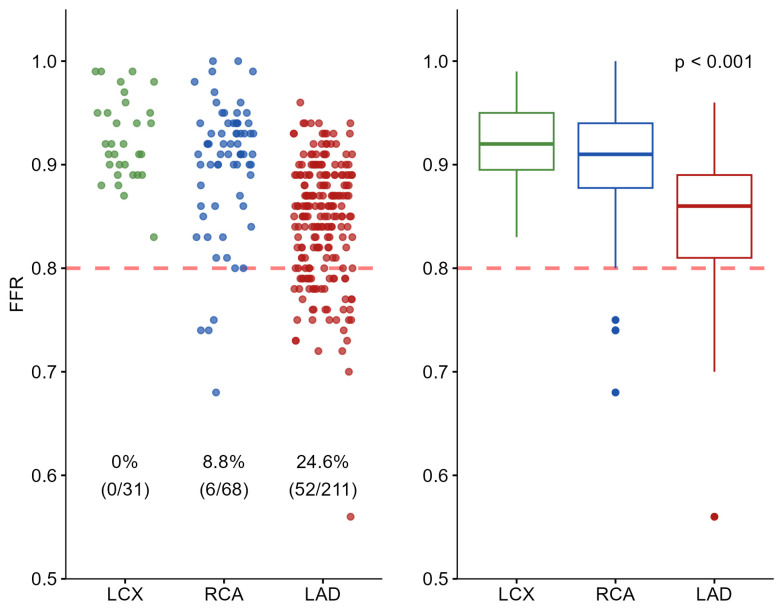
Scatter plot and boxplot of FFR values by coronary artery (N = 310). The LAD was more frequently assessed than the RCA and LCX and exhibited a higher rate of positive FFR values (≤0.80) compared with the other arteries (*p* < 0.001). The red dashed line indicates the FFR ischemic threshold of 0.80. LAD: Left Anterior Descending artery; LCX: Left Circumflex Artery; RCA: Right Coronary Artery.

The only variable identified in univariable analysis as being associated with FFR positivity was the coronary artery evaluated (*p* < 0.001). Positive FFR values were observed in 52 of the 211 LAD lesions (24.6%), in 6 of the 68 RCA lesions (8.8%), and in none of the 31 LCX lesions. Compared with the LAD, the probability of obtaining a positive FFR was significantly lower for the RCA (odds ratio 0.32, 95% confidence interval 0.12–0.70) and for the LCX (odds ratio 0.05, 95% confidence interval 0.01–0.35). No positive FFR values were observed in the LCX group. Therefore, odds ratio estimates for this subgroup were derived using Firth’s penalized logistic regression and should be interpreted with caution due to the absence of positive FFR. In the multivariable Firth’s penalized logistic regression model adjusting for vessel territory, age, sex, diabetes, hypertension, and smoking history, the coronary artery assessed remained an independent predictor of FFR positivity (RCA: adjusted OR 0.34, 95% CI 0.13–0.78, *p* = 0.009; LCX: adjusted OR 0.05, 95% CI 0.00–0.34, *p* < 0.001). Age was also independently associated with FFR results, with older patients showing a slightly lower likelihood of a positive FFR (adjusted OR 0.97, 95% CI 0.94–1.00, *p* = 0.035). No other covariate reached statistical significance. Figure 2 illustrates the distribution of FFR values according to coronary artery using scatterplots and boxplots. A significant difference in FFR values among the three coronary territories was demonstrated by the Kruskal–Wallis test (*p* < 0.001).

### 3.3. Clinical Outcomes

**Figure 3:**
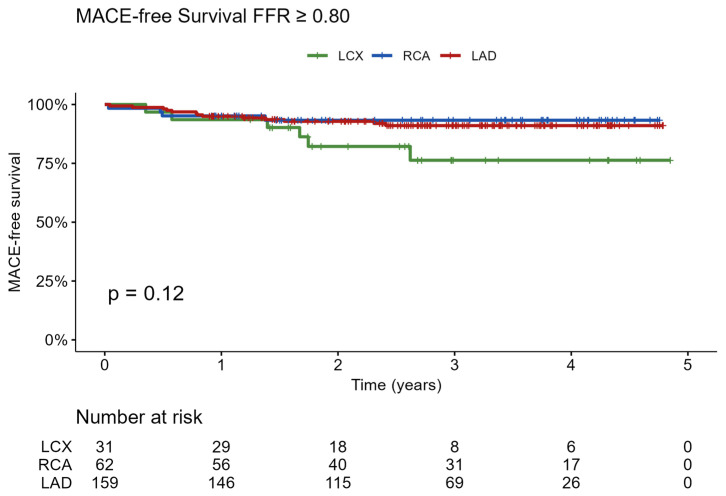
Kaplan–Meier curve of MACE-free survival in patients with negative FFR (>0.80) and deferred revascularization, stratified by studied artery (N = 252). Five-year MACE-free survival was 91.0% for the LAD, 93.3% for the RCA, and 76.3% for the LCX. Although the LCX showed a numerically lower survival, the log-rank test was not significant (*p* = 0.12), indicating no statistical difference between groups. LAD: Left Anterior Descending artery; LCX: Left Circumflex artery; RCA: Right Coronary Artery.

Among the 252 patients with negative FFR values and deferred coronary revascularization, long-term clinical outcomes were assessed over a follow-up period of up to five years. During this period, a total of 23 MACE occurred, including 13 events in the LAD group, 4 in the RCA group, and 6 in the LCX group. Five-year MACE-free survival was 91.0% in patients with LAD lesions, 93.3% in patients with RCA lesions, and 76.3% in patients with LCX lesions. Kaplan–Meier survival analysis did not demonstrate a statistically significant difference between the three groups (log-rank *p* = 0.12), as shown in Figure 3. In univariable Cox regression among FFR-negative patients, vessel territory was not a statistically significant predictor of MACE (overall *p* = 0.10). Compared to the LAD, neither the RCA (HR 0.80, 95% CI 0.26–2.46, *p* = 0.70) nor the LCX (HR 2.41, 95% CI 0.93–6.36, *p* = 0.074) reached statistical significance, although the LCX showed a non-significant trend toward a higher hazard of MACE.

## 4. Discussion

The present study demonstrates that the functional significance of angiographically intermediate coronary stenoses differs substantially according to the coronary artery evaluated. Lesions located in the left anterior descending artery were significantly more likely to be associated with ischemia, as assessed by fractional flow reserve, than lesions located in the right coronary artery or the left circumflex artery. Importantly, despite these inter-territorial physiological differences, patients for whom revascularization was deferred based on a negative FFR experienced similarly favorable long-term clinical outcomes, irrespective of the vessel involved. These findings provide novel insights into vessel-specific coronary physiology and support a more nuanced interpretation of intermediate coronary lesions in routine clinical practice.

### 4.1. Physiopathological Interpretation of Vessel-Specific FFR Differences

The most plausible explanation for the observed differences in FFR positivity among coronary arteries lies in the amount of myocardium subtended by each vessel. The LAD typically supplies the largest proportion of left ventricular myocardial mass, frequently exceeding 40%, whereas the RCA and LCX usually perfuse smaller territories depending on coronary dominance patterns. Experimental and clinical studies have consistently demonstrated that the volume of myocardium at risk is an independent determinant of FFR for a given degree of angiographic stenosis. Leone and colleagues showed that FFR values decrease in proportion to the myocardial mass supplied by the stenotic vessel, irrespective of lesion length or angiographic severity [8]. More recent data have reinforced this concept, demonstrating a relationship between coronary volume, myocardial mass, and FFR, including in post-PCI settings [9]. In parallel, imaging-based approaches using coronary computed tomography angiography and Voronoi modeling have confirmed that the LAD subtends the greatest myocardial territory in most patients [10]. Our findings are fully consistent with these observations and extend them to real-world clinical practice by demonstrating that intermediate LAD lesions are far more likely to reach ischemic thresholds than comparable lesions located in the RCA or LCX [11]. This phenomenon may also explain why angiographically similar stenoses can result in different physiological consequences depending on their anatomical location.

Beyond myocardial territory, anatomical and hydrostatic factors may further contribute to inter-vessel differences in measured FFR. The LAD predominantly supplies the anterior wall of the heart, whereas the RCA and LCX mainly perfuse inferior and posterior myocardial segments. When patients are examined in the supine position, vertical height differences between the coronary ostium and the distal pressure sensor may generate hydrostatic pressure gradients that influence distal coronary pressure measurements [12]. Prior studies have reported modest but measurable variations in FFR related to body position and coronary height relative to the aortic root [13]. However, these effects are likely clinically negligible in most patients and may only play a role in lesions with borderline FFR values.

Finally, microvascular function may also play a role in vessel-specific physiological behavior. Differences in microvascular resistance between coronary territories and between individuals could influence hyperemic flow and pressure gradients, thereby modulating FFR values. Although microvascular dysfunction was not systematically assessed in the present study, it remains an important potential confounder and should be considered in future investigations.

### 4.2. Clinical Implications and Prognostic Significance

The present findings have important clinical implications for the interpretation of intermediate coronary stenoses. They suggest that angiographic severity should not be interpreted uniformly across all coronary arteries. Moderate lesions in the RCA and LCX may require greater anatomical severity to become hemodynamically significant compared with lesions located in the LAD. Consequently, clinicians should adopt a vessel-specific physiological perspective when evaluating intermediate stenoses and avoid extrapolating LAD-derived expectations to all coronary territories without considering myocardial territory and anatomical context. 

Importantly, our findings do not support modifying established FFR decision thresholds. Current cut-offs remain valid across coronary territories. Rather, these results suggest that FFR values should be interpreted within a vessel-specific physiological context. In particular, borderline FFR values may warrant more nuanced interpretation depending on the coronary artery involved, taking into account myocardial territory and anatomical characteristics. This approach may help refine clinical decision-making without compromising the simplicity and robustness of current physiology-guided strategies.

In addition, our study provides reassuring prognostic information. Patients for whom revascularization was deferred based on a negative FFR experienced similarly favorable long-term outcomes regardless of the coronary artery evaluated. This observation reinforces the robustness and safety of physiology-guided decision-making and confirms that deferral of intervention based on negative FFR is appropriate not only in the LAD but also in the RCA and LCX. Although a numerically higher incidence of major adverse cardiovascular events was observed in the LCX group, this difference did not reach statistical significance and should be interpreted with caution given the small sample size of this subgroup. Larger multicenter studies will be required to determine whether subtle prognostic differences exist among coronary territories in patients with deferred lesions.

From a health-economic perspective, these results further support the use of coronary physiology to guide revascularization strategies. Multiple studies have demonstrated that FFR-guided approaches reduce unnecessary interventions while preserving clinical outcomes and lowering healthcare costs [14,15]. A refined understanding of vessel-specific coronary physiology may further improve the cost-effectiveness of invasive coronary assessment by optimizing the selection of lesions that truly require treatment.

## 5. Study Limitations

Several limitations should be acknowledged. First, the retrospective and single-center design of the study introduces the possibility of selection bias, as FFR measurements were performed only in lesions considered functionally uncertain based on visual angiographic assessment. Second, FFR assessments were conducted by multiple operators over a four-year period, which may have introduced inter-operator variability, although this reflects real-world clinical practice. In addition, no independent core laboratory adjudication of FFR measurements was performed, which may have introduced measurement variability and potential bias. The route of adenosine administration (intravenous or intracoronary) was left at the operator’s discretion and was not recorded on a per-patient basis, precluding a direct comparison of FFR values between the two modalities. Lesion location within the coronary tree (proximal vs. mid/distal segments), which may influence FFR values, was not systematically recorded in this retrospective dataset and could not be analyzed. Third, the relatively small number of LCX cases represents an important limitation, reducing the ability to detect outcome differences in this subgroup. This is further supported by a post hoc power analysis (observed power = 0.545), indicating that the study was underpowered for between-group comparisons. Finally, the absence of a control group managed solely based on angiographic assessment precludes direct comparison with anatomy-guided strategies. Despite these limitations, the present study provides novel and clinically relevant insights into vessel-specific coronary physiology in a real-world population.

## 6. Conclusions

FFR positivity among angiographically intermediate lesions varies according to the coronary territory, with LAD lesions more frequently reaching ischemic thresholds than those in the RCA or LCX. Despite these differences, deferral of revascularization based on a negative FFR was associated with similarly favorable long-term outcomes across all vessels. These findings support a vessel-specific physiological interpretation of intermediate stenoses, driven primarily by the myocardial territory supplied, while confirming the safety of FFR-guided deferral irrespective of coronary location.

## Figures and Tables

**Table 1:** Patient characteristics by FFR status (N = 310). Odds ratios and *p*-values from univariable analyses are reported. No clinical characteristics, risk factors, or treatments were significantly associated with a positive FFR. The only variable identified in univariable analysis as being associated with FFR positivity was the coronary artery assessed (*p* < 0.001). * No positive FFR results were observed in the LCX group. Odds ratio estimates were obtained using Firth’s penalized logistic regression and should be interpreted with caution. BSA: Body Surface Area; BMI: Body Mass Index; CABG: Coronary Artery Bypass Graft surgery; CKD: Chronic Kidney Disease; GFR: Glomerular Filtration rate; LAD: Left Anterior Descending; LCX: Left Circumflex Artery; MI: Myocardial Infarction; PCI: Percutaneous Coronary Intervention; RCA: Right Coronary Artery.

	Variable	Negative FFR (>0.80) n = 252 Mean ± SD N (%)	Positive FFR (≤0.80) n = 58 Mean ± SD N (%)	OR (CI 95%)	*p* Value	Adjusted OR (CI 95%)	*p* Value
Demographics	Age (years)	66.9 ± 9.5	64.3 ± 10.7	0.996 (0.99–1.00)	0.062	0.97 (0.94–1)	0.035
	Sex, M	176 (69.8)	44 (75.9)	1.36 (0.72–2.70)	0.36	1.34 (0.7–2.71)	0.38
	BSA (m^2^)	1.92 ± 0.20	1.92 ± 0.27	1.00 (0.82–1.22)	0.99		
	BMI (kg/m^2^)	27.2 ± 4.8	27.6 ± 4.9	1.00 (0.99–1.01)	0.53		
	Current smoker	87 (34.5)	14 (24.1)	0.60 (0.30–1.14)	0.13	0.57 (0.28–1.13)	0.11
Medical history	Diabetes	82 (32.5)	20 (34.5)	1.09 (0.59–1.97)	0.78	1.07 (0.55–2.04)	0.83
	Hypertension	186 (73.8)	43 (74.1)	1.02 (0.54–2.00)	0.96	1.15 (0.58–2.37)	0.70
	Hypercholesterolemia	201 (79.8)	48 (82.8)	1.22 (0.60–2.70)	0.61		
	Previous MI	56 (22.2)	12 (20.7)	0.91 (0.44–1.79)	0.80		
	Previous PCI	66 (26.2)	15 (25.9)	0.98 (0.50–1.85)	0.96		
	Previous CABG	8 (3.2)	1 (1.7)	0.53 (0.03–3.00)	0.56		
	Angina	117 (46.4)	32 (55.2)	1.42 (0.80–2.54)	0.23		
	Hemoglobin (g/dL)	14.0 ± 1.68	14.2 ± 1.66	1.01 (0.98–1.03)	0.538		
	Creatinine (mg/dL)	1.02 ± 0.34	0.98 ± 0.23	0.95 (0.83–1.08)	0.44		
	GFR (MDRD)	78.7 ± 24.6	81.7 ± 26.3	1.00 (0.99–1.01)	0.40		
	Chronic kidney disease	62 (24.6)	8(13.8)	0.49 (0.21–1.04)	0.08		
KDIGO Class (CKD)	I	66 (26.2)	15 (25.9)		0.87		
	II	137 (54.4)	34 (58.6)	1.08 (0.56–2.14)			
	III	44 (17.5)	9 (15.5)	0.92 (0.36–2.21)			
	IV–V	5 (2.0)	0 (0.0)	0.39 (0.01–3.75)			
LVEF	Normal (≥50%)	209 (82.9)	41 (81)				
	Mid-range (40–50%)	28 (11.1)	6 (10.3)	1.01 (0.37–2.37)			
	Reduced (<40%)	15 (6.0)	5 (8.6)	1.57 (0.52–4.14)	0.71		
Treatments	Aspirin	218 (86.5)	50 (86.2)	0.97 (0.44–2.38)	0.95		
	Beta blockers	149 (59.1)	34 (58.6)	0.94 (0.55–1.76)	0.94		
	ACEi—ARB	151 (59.9)	35 (60.3)	1.02 (0.57–1.84)	0.95		
	Statins	184 (73)	41 (70.7)	0.89 (0.48–1.71)	0.72		
	P2Y12	43 (17.1)	12 (20.7)	1.27 (0.6–2.53)	0.51		
Vessel	LAD	159 (63.1)	52 (89.7)		**<0.001**		
	RCA	62 (24.6)	6 (10.3)	0.32 (0.12–0.70)		0.34 (0.13–0.78)	0.009
	LCX	31 (12.3)	0 (0) *	0.05 (0.01–0.35)		0.05 (0–0.34)	<0.001

## Data Availability

The data underlying this study were derived from routinely collected clinical records and are not publicly available due to patient privacy and ethical restrictions. De-identified data may be made available from the corresponding author upon reasonable request and with permission from the institutional ethics committee.

## Abbreviations

BSA	Body surface area
BMI	Body mass index
CABG	Coronary artery bypass graft surgery
CKD	Chronic kidney disease
FFR	Fractional flow reserve
GFR	Glomerular filtration rate
LAD	Left anterior descending artery
LCX	Left circumflex artery
MACE	Major adverse cardiovascular events
MI	Myocardial infarction
PCI	Percutaneous coronary intervention
RCA	Right coronary artery
